# Supplementary material for: Evaluating variable selection methods for multivariable regression models: A simulation study protocol
Source: PLoS One. 2024 Aug 9;19(8):e0308543. doi: 10.1371/journal.pone.0308543 (PMC11315300; doi:10.1371/journal.pone.0308543)
Supplement: S1 Fig — (PDF) [file pone.0308543.s001.pdf]

S1 Fig. Correlation network graph.

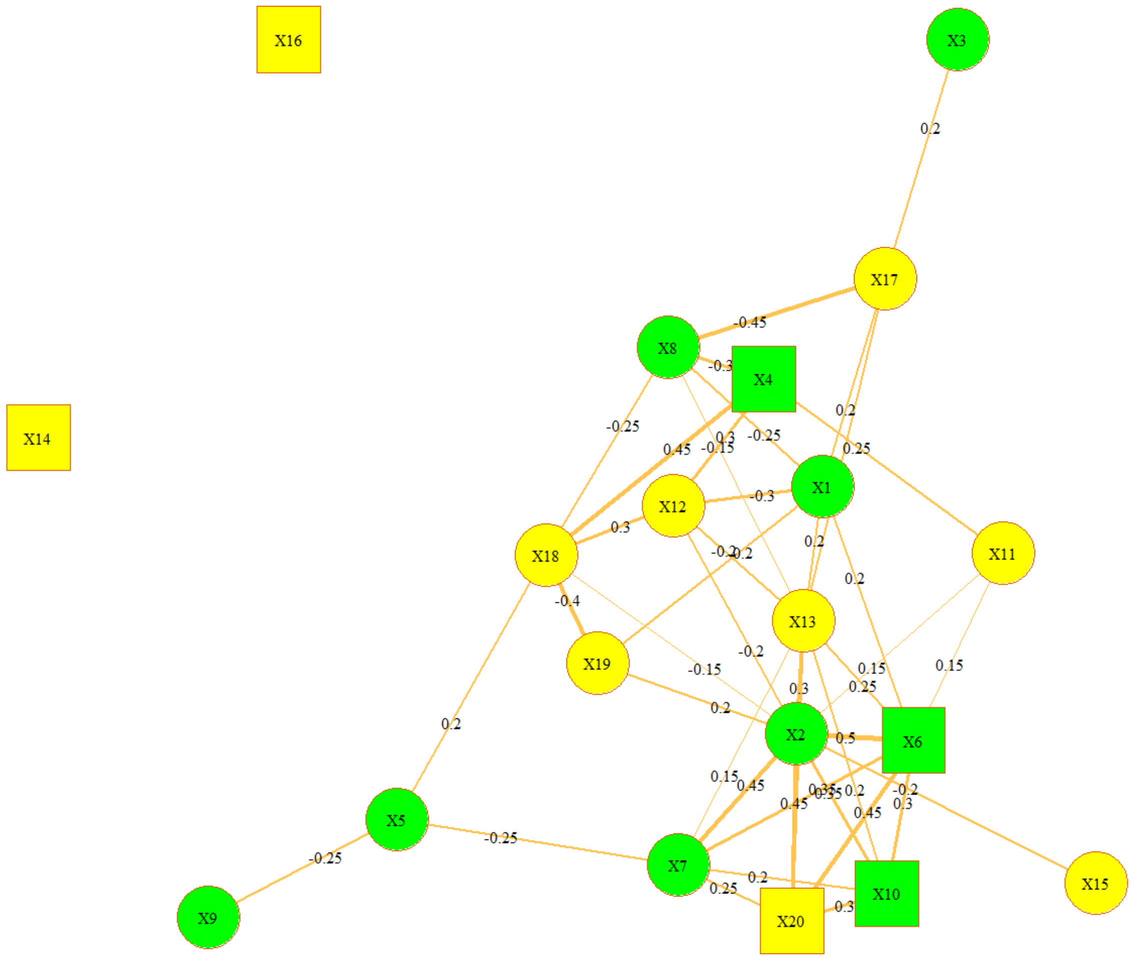

Fig S1. Correlation structure of predictors and noise variables. Predictors are green, noise variables are yellow. Binary variables are shown as boxes, continuous variables as circles. Widths of edges are proportional to correlation.
