# Supplementary material for: Evaluating variable selection methods for multivariable regression models: A simulation study protocol
Source: PLoS One. 2024 Aug 9;19(8):e0308543. doi: 10.1371/journal.pone.0308543 (PMC11315300; doi:10.1371/journal.pone.0308543)
Supplement: S2 Fig — (PDF) [file pone.0308543.s002.pdf]

S2 Fig. Absolute standardized regression coefficients plotted against coefficients of determination for each independent variable.

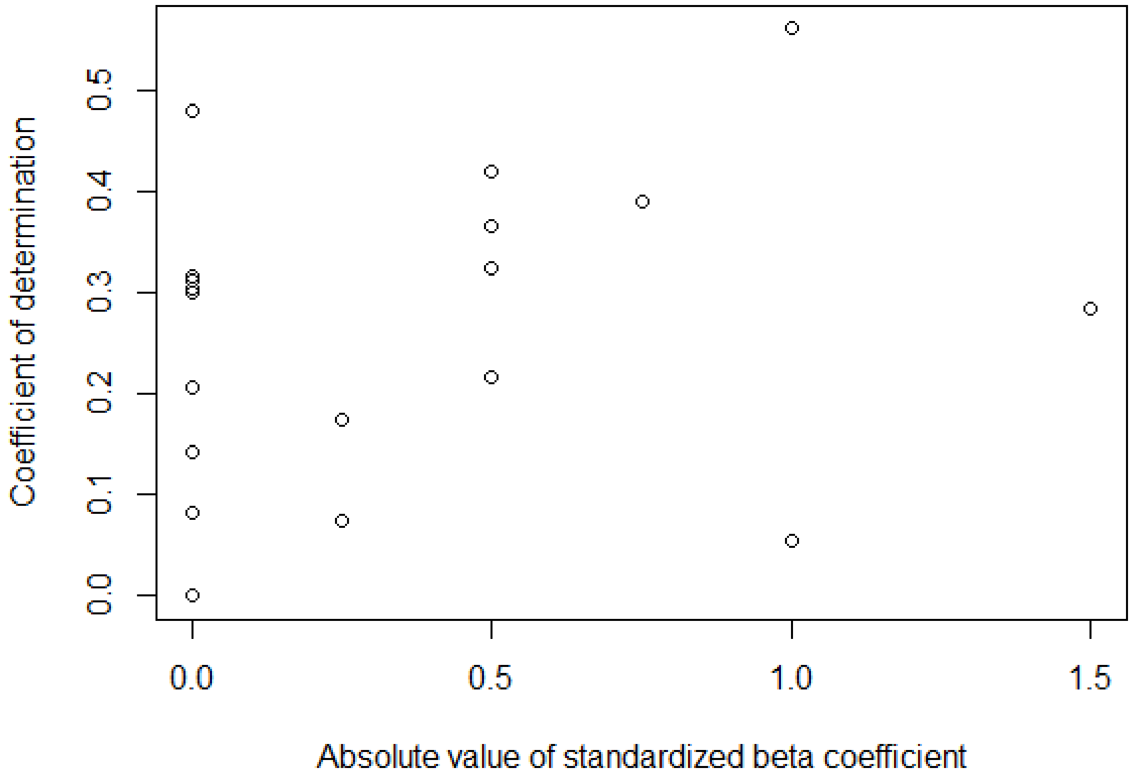

Fig S2. Absolute values $|\beta_j^{sd}|$ plotted against the coefficients of determination R_j^2 for the regression between X_j and all other variables $X_l, l = 1, \ldots, 20, l \neq j$.
