# Supplementary material for: Evaluating variable selection methods for multivariable regression models: A simulation study protocol
Source: PLoS One. 2024 Aug 9;19(8):e0308543. doi: 10.1371/journal.pone.0308543 (PMC11315300; doi:10.1371/journal.pone.0308543)
Supplement: S3 Fig — (PDF) [file pone.0308543.s003.pdf]

S3 Fig. Nonlinear effects.

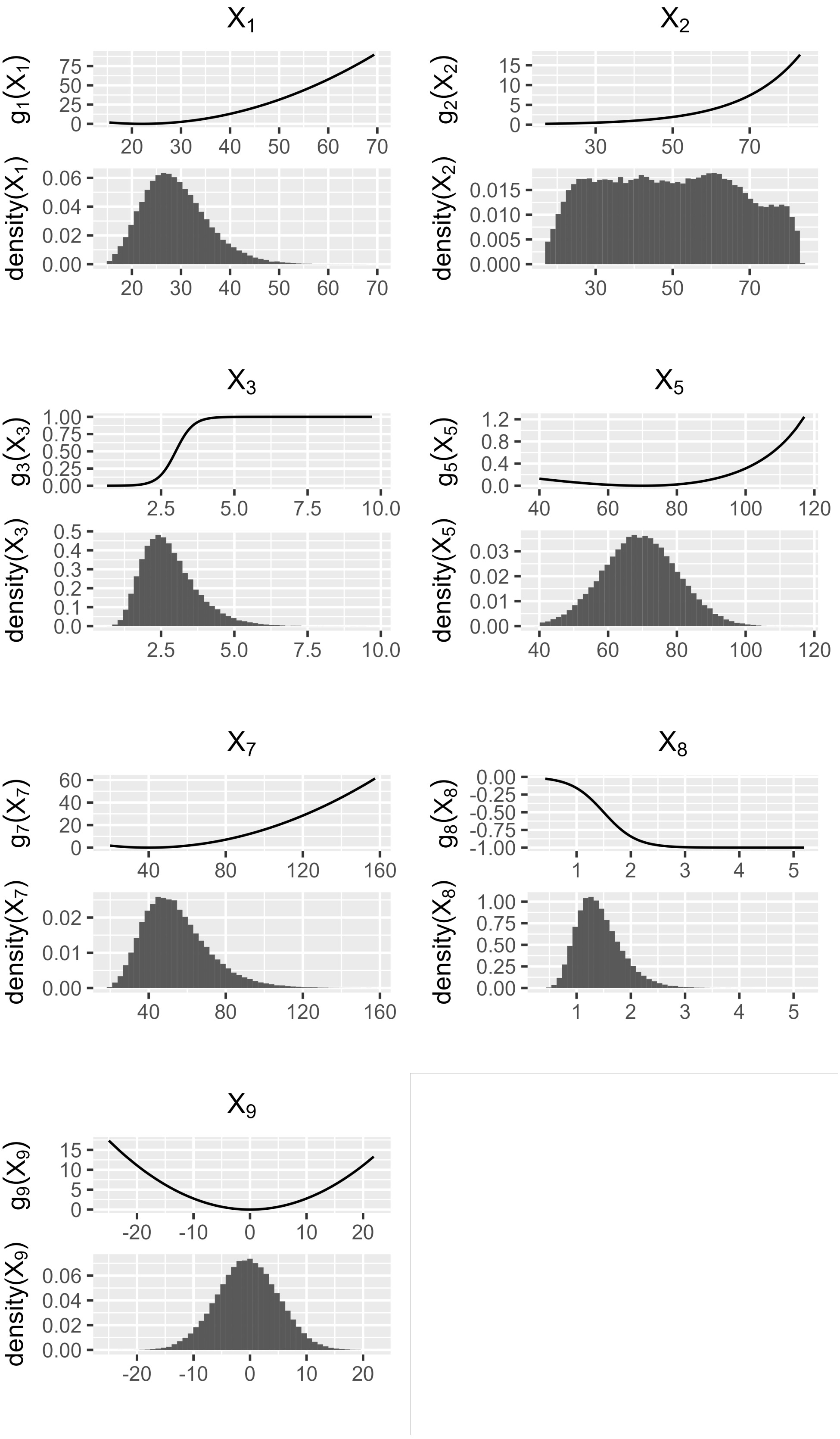

Fig S3. Functional forms of nonlinear effects of continuous predictors in settings 1b-7b. For each variable, the nonlinear function is shown above the distribution of the variable (histograms based on D_P).
