# Supplementary material for: Evaluating variable selection methods for multivariable regression models: A simulation study protocol
Source: PLoS One. 2024 Aug 9;19(8):e0308543. doi: 10.1371/journal.pone.0308543 (PMC11315300; doi:10.1371/journal.pone.0308543)
Supplement: S1 Appendix — (PDF) [file pone.0308543.s004.pdf]

S1: Details of the simulation design

Contents

S1.1 Simulation of independent variables (predictors and noise variables) 1
S1.2 Functional forms of simulated nonlinear effects 2
S1.3 Calculation of sample sizes for logistic regression 2
S1.4 Expected shrinkage factors 3
S1.5 Firth correction in logistic regression 4
S1.6 Performance measures 5

S1.1 Simulation of independent variables (predictors and noise variables)

Our choice of independent variables is inspired by a regression model reported by Sheppard et al. [1] for predicting the difference between diastolic blood pressure readings as measured ambulatory/at home vs. in the clinic. Sheppard et al. [1] included the following variables in their model: BMI (X_1), age (X_2), gender (X_4), diastolic blood pressure (X_5), taking medication against hypertension (yes/no, X_6), pulse pressure (X_7), the difference between the first and third diastolic blood pressure readings (X_9), and cardiovascular disease (yes/no, X_{10}). They also included previous diagnosis of hypertension (yes/no), but we did not choose this variable due to its very strong correlation with hypertension medication. Instead, we added LDL (X_3) and HDL (X_8) cholesterol to obtain ten predictors overall.
Moreover, we chose ten noise variables from NHANES data: married or living with partner (yes/no, X_{14}), smoking (yes/no, X_{16}), taking medication against high cholesterol (yes/no, X_{20}), and seven continuous variables ($X_{11}-X_{13}$, X_{15}, $X_{17}-X_{19}$) consisting of various blood test results. Each noise variable was chosen for at least one of the following reasons: a) the variable might be plausibly included in a cardiovascular regression model, b) the variable is correlated with the predictors, c) the variable helps to obtain a mixture of three binary and seven continuous noise variables, such that the distributions of the noise variables are comparable to those of the predictors.
To obtain the distributions from the NHANES data, we used the following strategies:

- For binary variables: fitting a Bernoulli distribution with probability p estimated from the frequency table (apart from gender, where p was set equal to 0.5 without considering the exact frequency in the data).
- For age (X_2): approximating the empirical cumulative distribution function (as neither a normal nor log-normal distribution is a suitable fit).

- For all other continuous variables: fitting either a normal or log-normal distribution, depending on which distribution appears to be a better fit based on visual inspection of the histogram.

For each continuous variable, we truncate its distribution with the minimum of the variable in the NHANES data as the lower bound and the maximum as the upper bound. This prevents unrealistic and extreme values, as well as numerical issues in the simulation.

S1.2 Functional forms of simulated nonlinear effects

The functions $g_j(x)$ for the nonlinear effects are defined as follows:

X_1: $g_1(x) = \left(\frac{x-22}{5}\right)^2$

X_2: $g_2(x) = \exp\left(\frac{x-40}{15}\right)$

X_3: $g_3(x) = \frac{1}{1+\exp(-(x-3)/0.3)}$

X_5: $g_5(x) = (\log(140-x) - \log(70))^2$

X_7: $g_7(x) = \left(\frac{x-40}{15}\right)^2$

X_8: $g_8(x) = \frac{-1}{1+\exp(-(x-1.5)/0.3)}$

X_9: $g_9(x) = \left(\frac{x}{6}\right)^2$

See Fig S3 for graphical depictions of the functions.

S1.3 Calculation of sample sizes for logistic regression

We describe our procedure for "aligning" the sample sizes $n_k^{(log)}$ for logistic regression to the sample sizes $n_k^{(lin)}$ for linear regression ($k = 1, \ldots, 7$), such that at sample size $n = n_k^{(log)}$, the regression coefficients in the logistic regression have approximately the same standard errors as the regression coefficients in the linear regression at $n = n_k^{(lin)}$. Let $j = 1, \ldots, 20$. The standard error of $\hat{\beta}_j$ can be written as a product of $\frac{1}{\sqrt{n}}$ and a part c_j that does not depend on n. This holds for both linear and logistic regression:

$$SE^{(lin,n)}(\hat{\beta}_j) = \frac{1}{\sqrt{n}} c_j^{(lin)} \rightarrow c_j^{(lin)} = \sqrt{n} SE^{(lin,n)}(\hat{\beta}_j) \tag{1}$$

$$SE^{(log,n)}(\hat{\beta}_j) = \frac{1}{\sqrt{n}} c_j^{(log)} \rightarrow c_j^{(log)} = \sqrt{n} SE^{(log,n)}(\hat{\beta}_j) \tag{2}$$

If samples sizes $n^{(lin)}, n^{(log)}$ are allowed to differ between linear and logistic regression, we have

$$SE^{(lin,n^{(lin)})}(\hat{\beta}_j) = SE^{(log,n^{(log)})}(\hat{\beta}_j)$$

if and only if

$$n^{(log)} = \left(\frac{c_j^{(log)}}{c_j^{(lin)}}\right)^2 n^{(lin)} \qquad (3)$$

Equation (3) can be used to calculate $n_1^{(log)}, \ldots, n_7^{(log)}$ for event rate 0.3 by plugging $n_1^{(lin)}, \ldots, n_7^{(lin)}$ in the right hand side. This requires estimating $c_j^{(lin)}, c_j^{(log)}$, which is done as follows. For $n = 6400$ (the largest sample size for linear regression; we choose this sample size to reduce the risk of separation in logistic regression), we simulate

- 5000 datasets for setting 1 for linear regression ($R^2 = 0.45$ and linear effects), and
- 5000 datasets for setting 4 for logistic regression (event rate 0.3, Cox-Snell $R^2_{CS} = 0.40$).

For each dataset, a global linear or logistic regression model is fitted, yielding 5000 point estimates for the coefficients for each setting. We estimate the standard errors (SEs) of the coefficients by taking the standard deviation over these point estimates. This yields vectors of estimated SEs

$$\widehat{SE}^{(lin,n)}(\hat{\beta}_1), \ldots, \widehat{SE}^{(lin,n)}(\hat{\beta}_{20}),$$

and

$$\widehat{SE}^{(log:0.3,n)}(\hat{\beta}_1), \ldots, \widehat{SE}^{(log:0.3,n)}(\hat{\beta}_{20}).$$

We then estimate $c_j^{(lin)}$ and $c_j^{(log:0.3)}$ using the formulas (1) and (2).
Finally, we can use formula (3) to calculate $n_k^{(log)}$ depending on $n_k^{(lin)}$. (Note: the fraction $c_j^{(lin)}/c_j^{(log)}$ is equal to $SE^{(lin,n)}(\hat{\beta}_j)/SE^{(log,n)}(\hat{\beta}_j)$, so we could also use the estimated SEs directly and omit calculating the constants.)
Depending on the variable j which is used in equation (3), different sets of sample sizes are obtained. We choose X_1 as the reference variable for calculating the sample sizes. X_1 has the largest effect size ($\beta_1^{sd} = 1.5$) and yields the largest required sample sizes for event rate 0.3.
While we could in principle use the above procedure to align sample sizes also for event rate 0.05, we have found that at low event rates, the procedure is rather sensitive to small changes in the input data (e.g., changes to the signs of the regression coefficients). Since formula (3) includes a quotient, even small absolute changes in $\widehat{SE}^{(log,n)}(\hat{\beta}_j)$ and $\widehat{SE}^{(lin,n)}(\hat{\beta}_j)$ can induce large relative changes in the value of the quotient $\left(\widehat{SE}^{(log,n)}(\hat{\beta}_j)/\widehat{SE}^{(lin,n)}(\hat{\beta}_j)\right)^2$.

S1.4 Expected shrinkage factors

Based on sample size and the known true R^2, we can calculate expected shrinkage factors for each setting, i.e., the factor by which estimated regression coefficients would have to

be multiplied in order to adjust for overfitting. The larger (i.e., closer to 1) the expected shrinkage factor, the smaller the overfitting potential.

For linear regression, we use the formula of Copas [2, 3]:

$$S_C = 1 + \frac{p-2}{n \ln(1-R^2)},$$

where p is the total number of variables (in our case, $p = 20$).

For logistic regression, we use the heuristic shrinkage factor of Van Houwelingen and Le Cessie [4, 5]:

$$S_{VH} = 1 + \frac{p}{n \ln(1-R^2_{CS})}.$$

Here, R^2_{CS} denotes the Cox-Snell R^2.

The resulting shrinkage factors are reported in Table S1.1. Note that the shrinkage factor for linear regression with $R^2 = 0.15$ and sample size $n = 100$ is negative. Indeed, using the formulas above can result in negative values. In this case, when performing post-estimation shrinkage in practice, one would set the shrinkage factor to zero (i.e., predict the overall mean or overall observed risk) [2].

Table S1.1. Expected shrinkage factors for linear and logistic regression

linear regression	n	100	200	400	*500*	800	1600	3200	6400
$R^2 = 0.45$ (settings 1, 1b)	S_C	0.70	0.85	0.92	0.94	0.96	0.98	0.99	1.00
$R^2 = 0.15$ (settings 2, 2b)	S_C	-0.11	0.45	0.72	0.78	0.86	0.93	0.97	0.98
$R^2 = 0.7$ (settings 3, 3b)	S_C	0.85	0.93	0.96	0.97	0.98	0.99	1.00	1.00
logistic regression, event rate 0.3	n	183	365	730	*1667*	1461	2922	5844	11,687
$R^2_{CS} = 0.40$ (setting 4)	S_{VH}	0.79	0.89	0.95	0.98	0.97	0.99	0.99	1.00
$R^2_{CS} = 0.43$ (setting 4b)	S_{VH}	0.81	0.90	0.95	0.98	0.98	0.99	0.99	1.00
$R^2_{CS} = 0.13$ (setting 5)	S_{VH}	0.22	0.61	0.80	0.91	0.90	0.95	0.98	0.99
$R^2_{CS} = 0.14$ (setting 5b)	S_{VH}	0.28	0.64	0.82	0.92	0.91	0.95	0.98	0.99
logistic regression, event rate 0.05	n	2000	4000	8000	*10,000*	– –	– –	– –	– –
$R^2_{CS} = 0.16$ (setting 6)	S_{VH}	0.94	0.97	0.99	0.99	–	–	–	–
$R^2_{CS} = 0.20$ (setting 6b)	S_{VH}	0.96	0.98	0.99	0.99	–	–	–	–
$R^2_{CS} = 0.05$ (setting 7)	S_{VH}	0.81	0.90	0.95	0.96	–	–	–	–
$R^2_{CS} = 0.07$ (setting 7b)	S_{VH}	0.86	0.93	0.97	0.97	–	–	–	–

S1.5 Firth correction in logistic regression

We check each simulated dataset for separation to decide whether applying the Firth correction is necessary. First of all, we note that it suffices to check the global model: if separation occurs for a model with less variables selected, it must necessarily also

occur for the global model. For each simulation setting, before the repetitions of the main simulation start, we first simulate $n_{sim,firth}$ large datasets with sample size $n = 15000$. We assume that at this sample size, no separation occurs. We estimate the coefficient standard errors $SE^{(log,n=15000)}(\hat{\beta}_j)$ by taking the standard deviation over the point estimates in the $n_{sim,firth}$ repetitions. We then calculate the "expected" SEs for other sample sizes n (provided no separation occurs):

$$SE^{(expected,n)}(\hat{\beta}_j) = \widehat{SE}^{(log,15000)}(\hat{\beta}_j)\sqrt{15000/n},$$

where we have used that the SEs scale with $1/\sqrt{n}$.
Moreover, we calculate the ratio r_j as

$$r_j = \max(ModSE^{(n=15000)}(\hat{\beta}_j))/\widehat{SE}^{(log,15000)}(\hat{\beta}_j)$$

i.e., the ratio of the maximum model SE in the $n_{sim,firth}$ repetitions divided by $\widehat{SE}^{(log,15000)}(\hat{\beta}_j)$.
After this process has concluded, the actual simulation of the datasets begins. For each simulated dataset, we check whether the model SE of $\hat{\beta}_j$ is larger than

$$10 \times r_j \times SE^{(expected,n)}(\hat{\beta}_j) = 10 \times \max(ModSE^{(n=15000)}(\hat{\beta}_j)) \times \sqrt{15000/n}$$

This check is only performed for the binary predictors X_4, X_6 and X_{10}.
When applying the Lasso methods (Lasso, Relaxed Lasso, Adaptive Lasso), 10-fold cross-validation is performed and models are fitted on subsamples of the whole datasets. It is possible that separation occurs for one of these subsamples, while it does not occur for the whole dataset. Therefore, it does not suffice to check the whole dataset. Let D denote the simulated dataset in one of repetitions. After obtaining folds $D^{(1)}, \dots, D^{(10)}$, we check for separation on all sets $D \setminus D^{(k)}, k = 1, \dots, 10$. To save computation time during the check for separation, we choose identical CV fold identifiers for all three Lasso methods.

S1.6 Performance measures

Formulas for the performance measures are given in Tables S1.1 and S1.2 (for regression coefficients as estimands), Table S1.3 (for model selection as target), and Table S1.4 (for prediction as target). Here we explain some details about the formulas.

General notation The covariates $\boldsymbol{X} = (X_1, \dots, X_{20})$ take values in a space $\mathcal{X} \subset \mathbb{R}^{20}$, and the response variable Y takes values in $\mathcal{Y}$ (where $\mathcal{Y} = \mathbb{R}$ for linear regression, and $\mathcal{Y} = \{0, 1\}$ for logistic regression). Covariates $\boldsymbol{X}$ and response Y follow a joint distribution P on $\mathcal{X} \times \mathcal{Y}$. Note that each simulation setting corresponds to a distribution P from which the simulated dataset is drawn.

Performance measure	Definition	Approximation by simulation
Bias towards 0 (>0) or away from 0 (<0) ($\star$)		
a) unconditional	$E_{P^n}\left[\left(\hat{\beta}_j - \beta_j\right)\operatorname{sign}(\beta_j)\right]$	$\frac{1}{n_{sim}}\sum_{i=1}^{n_{sim}}\left(\hat{\beta}_j^{(i)} - \beta_j\right)\operatorname{sign}(\beta_j)$
b) conditional	$E_{P^n}\left[\left(\hat{\beta}_j - \beta_j\right)\operatorname{sign}(\beta_j) \mid \hat{\beta}_j \neq 0\right]$	$\frac{1}{n_{cond(j)}}\sum_{i=1}^{n_{cond(j)}}\left(\hat{\beta}_j^{(i)} - \beta_j\right)\operatorname{sign}(\beta_j)$
RMSE$\cdot\sqrt{n}$ ($\star$)		
a) unconditional	$\sqrt{nE_{P^n}\left[\left(\hat{\beta}_j - \beta_j\right)^2\right]}$	$\sqrt{\frac{n}{n_{sim}}\sum_{i=1}^{n_{sim}}\left(\hat{\beta}_j^{(i)} - \beta_j\right)^2}$
b) conditional	$\sqrt{nE_{P^n}\left[\left(\hat{\beta}_j - \beta_j\right)^2 \mid \hat{\beta}_j \neq 0\right]}$	$\sqrt{\frac{n}{n_{cond(j)}}\sum_{i=1}^{n_{cond(j)}}\left(\hat{\beta}_j^{(i)} - \beta_j\right)^2}$
Coverage probability of the 95% CI ($\star$)		
a) unconditional	$P^n\left(\beta_j \in \left[\widehat{CI}_{j,lower}, \widehat{CI}_{j,upper}\right]\right)$	$\frac{1}{n_{sim}}\sum_{i=1}^{n_{sim}} I\left(\beta_j \in \left[\widehat{CI}_{j,lower}^{(i)}, \widehat{CI}_{j,upper}^{(i)}\right]\right)$
b) conditional	$P^n\left(\beta_j \in \left[\widehat{CI}_{j,lower}, \widehat{CI}_{j,upper}\right] \mid \hat{\beta}_j \neq 0\right)$	$\frac{1}{n_{cond(j)}}\sum_{i=1}^{n_{cond(j)}} I\left(\beta_j \in \left[\widehat{CI}_{j,lower}^{(i)}, \widehat{CI}_{j,upper}^{(i)}\right]\right)$

Table S1.1. Performance measures for regression coefficients, part 1. Notation:
n_{sim}: Number of simulation repetitions. $i = 1, \ldots, n_{sim}$: indexes the repetitions of the simulation.
$\hat{\beta}_j$: estimate for β_j. $\widehat{CI}_{j,lower}$, $\widehat{CI}_{j,upper}$: lower and upper boundaries of the estimated 95% confidence interval for β_j.
$\hat{\beta}_j^{(i)}$: estimate for β_j in the i-th simulated dataset. $\widehat{CI}_{j,lower}^{(i)}$, $\widehat{CI}_{j,upper}^{(i)}$: lower and upper boundaries of the 95% CI for β_j estimated in the i-th simulated dataset.
($\star$): Calculation conditionally or unconditionally on selection is possible. For measures unconditional on selection, $\hat{\beta}_j^{(i)}$, $\widehat{CI}_{j,lower}^{(i)}$ and $\widehat{CI}_{j,upper}^{(i)}$ are set to 0 if the j-th variable was not selected. To calculate measures conditional on selection, only simulation repetitions are taken into account where $\hat{\beta}_j^{(i)} \neq 0$:
$n_{cond(j)} = \#\left\{i = 1, \ldots, n_{sim} \mid \hat{\beta}_j^{(i)} \neq 0\right\}$.

Performance measure	Definition	Approximation by simulation
Expected width of the 95% CI $\cdot\sqrt{n}$ ($\star$)		
a) unconditional	$\sqrt{n}E_{P^n}\left[\widehat{CI}_{j,upper} - \widehat{CI}_{j,lower}\right]$	$\frac{\sqrt{n}}{n_{sim}}\sum_{i=1}^{n_{sim}}\left(\widehat{CI}_{j,upper}^{(i)} - \widehat{CI}_{j,lower}^{(i)}\right)$
b) conditional	$\sqrt{n}E_{P^n}\left[\widehat{CI}_{j,upper} - \widehat{CI}_{j,lower} \mid \hat{\beta}_j \neq 0\right]$	$\frac{\sqrt{n}}{n_{cond(j)}}\sum_{i=1}^{n_{cond(j)}}\left(\widehat{CI}_{j,upper}^{(i)} - \widehat{CI}_{j,lower}^{(i)}\right)$
Type 1 error rate/Power ($\star$)		
a) unconditional	$P^n\left(0 \notin \left[\widehat{CI}_{j,lower}, \widehat{CI}_{j,upper}\right]\right)$	$\frac{1}{n_{sim}}\sum_{i=1}^{n_{sim}} I\left(0 \notin \left[\widehat{CI}_{j,lower}^{(i)}, \widehat{CI}_{j,upper}^{(i)}\right]\right)$
b) conditional	$P^n\left(0 \notin \left[\widehat{CI}_{j,lower}, \widehat{CI}_{j,upper}\right] \mid \hat{\beta}_j \neq 0\right)$	$\frac{1}{n_{cond(j)}}\sum_{i=1}^{n_{cond(j)}} I\left(0 \notin \left[\widehat{CI}_{j,lower}^{(i)}, \widehat{CI}_{j,upper}^{(i)}\right]\right)$
False positive rate/True positive rate	$P^n\left(\hat{\beta}_j \neq 0\right)$	$\frac{1}{n_{sim}}\sum_{i=1}^{n_{sim}} I\left(\hat{\beta}_j^{(i)} \neq 0\right)$
Kendall's τ_B for variable rankings	$E_{P^n}\left[\tau_B\left(\hat{\boldsymbol{\beta}}, \boldsymbol{\beta}\right)\right]$	$\frac{1}{n_{sim}}\sum_{i=1}^{n_{sim}} \tau_B\left(\hat{\boldsymbol{\beta}}^{(i)}, \boldsymbol{\beta}\right)$

Table S1.2. Performance measures for regression coefficients, part 2. Notation:
n_{sim}: Number of simulation repetitions. $i = 1, \ldots, n_{sim}$: indexes the repetitions of the simulation.
$\hat{\beta}_j$: estimate for β_j. $\widehat{CI}_{j,lower}$, $\widehat{CI}_{j,upper}$: lower and upper boundaries of the estimated 95% confidence interval for β_j.
$\hat{\beta}_j^{(i)}$: estimate for β_j in the i-th simulated dataset. $\widehat{CI}_{j,lower}^{(i)}$, $\widehat{CI}_{j,upper}^{(i)}$: lower and upper boundaries of the 95% CI for β_j estimated in the i-th simulated dataset.
($\star$): Calculation conditionally or unconditionally on selection is possible. For measures unconditional on selection, $\hat{\beta}_j^{(i)}$, $\widehat{CI}_{j,lower}^{(i)}$ and $\widehat{CI}_{j,upper}^{(i)}$ are set to 0 if the j-th variable was not selected. To calculate measures conditional on selection, only simulation repetitions are taken into account where $\hat{\beta}_j^{(i)} \neq 0$:
$n_{cond(j)} = \#\left\{i = 1, \ldots, n_{sim} \mid \hat{\beta}_j^{(i)} \neq 0\right\}$.

Notation in Tables S1.1 and S1.2 (regression coefficients as estimands) Performance measures include bias and RMSE$\cdot\sqrt{n}$ of the coefficients, the coverage and width$\cdot\sqrt{n}$ of the 95% confidence intervals, the type 1 error rate/power, the false positive rate/true positive rate, and Kendall's τ_B for variable rankings. Bias is multiplied by the sign of the true coefficient, such that positive values indicate bias towards zero and negative values indicate bias away from zero. E_{P^n} denotes the expectation over a sample with n i.i.d. observations, each distributed according to P. In this type of expectation, $\hat{\beta}_j$ denotes an estimate for β_j estimated from a sample with n observations. Analogously, $\widehat{CI}_j$ denotes a confidence interval for β_j estimated from a sample with n observations. The expectation E_{P^n} can be approximated by simulation, namely by averaging over n_{sim} simulated datasets with sample size n drawn from P^n.

For bias and RMSE$\cdot\sqrt{n}$ of coefficients, coverage and width$\cdot\sqrt{n}$ of confidence intervals, and type 1 error/power for variables, the measures can be calculated unconditionally or conditionally on selection. In the unconditional approach, the coefficients and their confidence limits for non-selected variables are set to zero. In the conditional approach, only simulation runs are taken into account where the specific variable is selected.

For better comparison, all regression coefficients were standardized for the calculation of bias and RMSE$\cdot\sqrt{n}$ of coefficients, width$\cdot\sqrt{n}$ of the CI, and Kendall's τ_B. This is suppressed in the notation of Tables S1.1 and S1.2.

For settings with nonlinear effects, β_j denotes one of the two possible estimands: $\beta_j^{(proj)}$ obtained by projection or $\beta_j^{(AS)}$ based on average slope.

**Performance measure**	**Definition**	**Approximation by simulation**
Selection rate of the true model	$E_{P^n}\left[\prod_{l\in L} I\left(\hat{\beta}_l \neq 0\right)\prod_{l'\in L'} I\left(\hat{\beta}_l = 0\right)\right]$	$\frac{1}{n_{sim}}\sum_{i=1}^{n_{sim}}\prod_{l\in L} I\left(\hat{\beta}_l^{(i)} \neq 0\right)\prod_{l'\in L'} I\left(\hat{\beta}_l^{(i)} = 0\right)$
Selection rate of an over-selection model	$E_{P^n}\left[I\left(\exists l' \in L' : \hat{\beta}_{l'} \neq 0\right)\prod_{l\in L} I\left(\hat{\beta}_l \neq 0\right)\right]$	$\frac{1}{n_{sim}}\sum_{i=1}^{n_{sim}} I\left(\exists l' \in L' : \hat{\beta}_{l'}^{(i)} \neq 0\right)\prod_{l\in L} I\left(\hat{\beta}_l^{(i)} \neq 0\right)$
Selection rate of an under-selection model	$P^n\left(\exists l \in L : \hat{\beta}_l = 0\right)$	$\frac{1}{n_{sim}}\sum_{i=1}^{n_{sim}} I\left(\exists l \in L : \hat{\beta}_l^{(i)} = 0\right)$

Table S1.3. Performance measures for model selection. Notation:
n_{sim}: Number of simulation repetitions. $i = 1, \ldots, n_{sim}$: indexes the repetitions of the simulation.
$\hat{\beta}_j$: estimate for β_j.
$\hat{\beta}_j^{(i)}$: estimate for β_j in the i-th simulated dataset.
$L = \{j : \beta_j \neq 0\}$: indices of true predictors, $L' = \{j : \beta_j = 0\}$: indices of noise variables.

Notation in Table S1.3 (model selection as target) The performance measures include the selection rate of the *true* model consisting exactly of the ten predictors, the selection rate of any *over-selection* model including all predictors as well as at least one noise variable, and the selection rate of any *under-selection* model not containing all predictors but possibly including noise variables. The same notation as in Tables S1.1 and S1.2 applies. Moreover, the set $L = \{j : \beta_j \neq 0\} = \{1, \ldots, 10\}$ denotes the indices of the predictors, and $L' = \{j : \beta_j = 0\} = \{11, \ldots, 20\}$ the indices of the noise variables.

Performance measure	Definition	Approximation by simulation
Local bias	$E_{P^n}\left[\boldsymbol{X}\left(\hat{\boldsymbol{\beta}}-\boldsymbol{\beta}\right) \mid \boldsymbol{X}\boldsymbol{\beta}\right]$	$\frac{1}{n_{sim}}\sum_{i=1}^{n_{sim}}\left(\boldsymbol{x}_k\left(\hat{\boldsymbol{\beta}}^{(i)}-\boldsymbol{\beta}\right)\right)$
Local RMSE $\cdot\sqrt{n}$	$\sqrt{nE_{P^n}\left[\left(\boldsymbol{X}\left(\hat{\boldsymbol{\beta}}-\boldsymbol{\beta}\right)\right)^2 \mid \boldsymbol{X}\boldsymbol{\beta}\right]}$	$\sqrt{\frac{n}{n_{sim}}\sum_{i=1}^{n_{sim}}\left(\boldsymbol{x}_k\left(\hat{\boldsymbol{\beta}}^{(i)}-\boldsymbol{\beta}\right)\right)^2}$
Global RMSE $\cdot\sqrt{n}$	$\sqrt{nE_{P^n}\left[E_P\left[\left(\boldsymbol{X}\left(\hat{\boldsymbol{\beta}}-\boldsymbol{\beta}\right)\right)^2\right]\right]}$	$\sqrt{\frac{n}{n_{sim}}\sum_{i=1}^{n_{sim}}\frac{1}{n_{test}}\sum_{k=1}^{n_{test}}\left(\boldsymbol{x}_k\left(\hat{\boldsymbol{\beta}}^{(i)}-\boldsymbol{\beta}\right)\right)^2}$
Global MAE	$E_{P^n}\left[F^{-1}_{\lvert\boldsymbol{X}(\hat{\boldsymbol{\beta}}-\boldsymbol{\beta})\rvert}(0.5)\right]$	$\frac{1}{n_{sim}}\sum_{i=1}^{n_{sim}}\mathrm{mdn}_{k=1,\ldots,n_{test}}\lvert\boldsymbol{x}_k\left(\hat{\boldsymbol{\beta}}^{(i)}-\boldsymbol{\beta}\right)\rvert$
AUC (for logistic regression)	$E_{P^n}\left[E_{P^2}\left[I\left(\hat{p}_1>\hat{p}_2\right)\lvert Y_1=1,Y_2=0\right]\right]$	$\frac{1}{n_{sim}}\sum_{i=1}^{n_{sim}}\frac{1}{\lvert M\rvert}\sum_{(k,k')\in M}I\left(\hat{p}_k^{(i)}>\hat{p}_{k'}^{(i)}\right)$ with $M=\{(k,k'):y_k=1,y_{k'}=0\}$
Integrated Calibration Index (ICI) for linear regression	$E_{P^n}\left[E_P\left[\lvert\hat{c}\left(\hat{Y}\right)-\hat{Y}\rvert\right]\right]$	$\frac{1}{n_{sim}}\sum_{i=1}^{n_{sim}}\frac{1}{n_{test}}\sum_{k=1}^{n_{test}}\lvert\hat{c}^{(i)}\left(\hat{y}_k^{(i)}\right)-\hat{y}_k^{(i)}\rvert$
Integrated Calibration Index (ICI) for logistic regression	$E_{P^n}\left[E_P\left[\lvert\hat{c}\left(\hat{p}\right)-\hat{p}\rvert\right]\right]$	$\frac{1}{n_{sim}}\sum_{i=1}^{n_{sim}}\frac{1}{n_{test}}\sum_{k=1}^{n_{test}}\lvert\hat{c}^{(i)}\left(\hat{p}_k^{(i)}\right)-\hat{p}_k^{(i)}\rvert$

Table S1.4. Performance measures for prediction. Notation:
n_{sim}: Number of simulation repetitions. $i=1,\ldots,n_{sim}$: indexes the repetitions of the simulation.
$\hat{\boldsymbol{\beta}}$: estimate for coefficient vector $\boldsymbol{\beta}$.
$\hat{\boldsymbol{\beta}}^{(i)}$: estimate for coefficient vector $\boldsymbol{\beta}$ in the i-th simulated dataset.
$(\boldsymbol{X},Y)$: observation vector and outcome of an observation in the test set
$\hat{Y}$: estimate for the outcome value Y based on the model fitted on the training set.
$(\boldsymbol{x}_k,y_k)$: observation vector and outcome of the k-th observation in the simulated test set, $k=1,\ldots,n_{test}$.
$\hat{y}_k^{(i)}$: estimate for the outcome value y_k of the k-th observation in the simulated test set based on the model of the i-th simulated training dataset.
For linear regression, it holds that $\hat{Y}=\boldsymbol{X}\hat{\boldsymbol{\beta}}$, $\hat{y}_k^{(i)}=\boldsymbol{x}_k\hat{\boldsymbol{\beta}}^{(i)}$.
For logistic regression, $\hat{p}$ denotes the estimated probability score for an observation $(\boldsymbol{X},Y)$ in the test set, and $\hat{p}_k^{(i)}$ the estimated probability for test observation $(\boldsymbol{x}_k,y_k)$ based on the model fitted on the i-th simulated training set.
$\hat{c}\left(\hat{Y}\right)$: value of the calibration curve at point $\hat{Y}$ (linear regression), $\hat{c}\left(\hat{p}\right)$: value of the calibration curve at point $\hat{p}$ (logistic regression), $\hat{c}^{(i)}\left(\hat{y}_k^{(i)}\right)$: value of the calibration curve at point $\hat{y}_k^{(i)}$ (linear regression), $\hat{c}^{(i)}\left(\hat{p}_k^{(i)}\right)$: value of the calibration curve at point $\hat{p}_k^{(i)}$ (logistic regression) based on the model of the i-th simulated dataset.

Notation in Table S1.4 (prediction) As before, E_{P^n} denotes the expectation over a (training) sample with n i.i.d. observations distributed according to P. Additionally, the expectation E_P denotes the expectation over a test observation $(\boldsymbol{X}, Y)$ distributed according to P. This expectation is approximated by averaging over n_{test} observations drawn i.i.d. from P. In the double expectation $E_{P^n}[E_P[\ldots]]$, $(\boldsymbol{X}, Y)$ denotes a test observation drawn from P, and $\hat{Y}$ (resp. $\hat{p}$) denotes the prediction for outcome Y (resp. the predicted probability for the outcome) based on the model fitted on a training sample with n observations drawn from P^n.

For the global MAE, $F^{-1}_{|\boldsymbol{X}(\hat{\boldsymbol{\beta}}-\boldsymbol{\beta})|}$ denotes the quantile function of the cumulative distribution function of $|\boldsymbol{X}(\hat{\boldsymbol{\beta}} - \boldsymbol{\beta})|$ (random variable w.r.t. sampling from the test data). The expression $\text{mdn}_{k=1,\ldots,n_{test}}$ denotes the median over the n_{test} observations in the test set.

For the AUC, the expectation E_{P^2} denotes the expectation over two test observations $(\boldsymbol{X}_1, Y_1), (\boldsymbol{X}_2, Y_2)$ drawn i.i.d. from P.

References

1. Sheppard JP, Stevens R, Gill P, Martin U, Godwin M, Hanley J, et al. Predicting Out-of-Office Blood Pressure in the Clinic (PROOF-BP): Derivation and Validation of a Tool to Improve the Accuracy of Blood Pressure Measurement in Clinical Practice. Hypertension. 2016;67(5):941–950.

2. Copas JB. Regression, prediction and shrinkage. Journal of the Royal Statistical Society Series B: Statistical Methodology. 1983;45(3):311–335.

3. Riley RD, Snell KI, Ensor J, Burke DL, Harrell Jr FE, Moons KG, et al. Minimum sample size for developing a multivariable prediction model: Part I–Continuous outcomes. Statistics in Medicine. 2019;38(7):1262–1275.

4. Van Houwelingen J, Le Cessie S. Predictive value of statistical models. Statistics in Medicine. 1990;9(11):1303–1325.

5. Riley RD, Snell KI, Ensor J, Burke DL, Harrell Jr FE, Moons KG, et al. Minimum sample size for developing a multivariable prediction model: PART II-binary and time-to-event outcomes. Statistics in Medicine. 2019;38(7):1276–1296.
