# Supplementary material for: Evaluating variable selection methods for multivariable regression models: A simulation study protocol
Source: PLoS One. 2024 Aug 9;19(8):e0308543. doi: 10.1371/journal.pone.0308543 (PMC11315300; doi:10.1371/journal.pone.0308543)
Supplement: S1 Table — (PDF) [file pone.0308543.s005.pdf]

S1 Table. Correlation table.

	X_1	X_2	X_3	X_4	X_5	X_6	X_7	X_8	X_9	X_{10}	X_{11}	X_{12}	X_{13}	X_{14}	X_{15}	X_{16}	X_{17}	X_{18}	X_{19}	X_{20}
X_1	100	0	0	0	0	20	0	-25	0	0	0	-30	20	0	0	0	20	0	-20	0
X_2	0	100	0	0	0	50	45	0	0	35	15	-20	30	0	-20	0	0	-15	20	45
X_3	0	0	100	0	0	0	0	0	0	0	0	0	0	0	0	0	20	0	0	0
X_4	0	0	0	100	0	0	0	-30	0	0	25	30	0	0	0	0	0	45	0	0
X_5	0	0	0	0	100	0	-25	0	-25	0	0	0	0	0	0	0	0	20	0	0
X_6	20	50	0	0	0	100	35	0	0	30	15	0	25	0	0	0	0	0	0	45
X_7	0	45	0	0	-25	35	100	0	0	20	0	0	15	0	0	0	0	0	0	25
X_8	-25	0	0	-30	0	0	0	100	0	0	0	0	-15	0	0	0	-45	-25	0	0
X_9	0	0	0	0	-25	0	0	0	100	0	0	0	0	0	0	0	0	0	0	0
X_{10}	0	35	0	0	0	30	20	0	0	100	0	0	20	0	0	0	0	0	0	30
X_{11}	0	15	0	25	0	15	0	0	0	0	100	0	0	0	0	0	0	0	0	0
X_{12}	-30	-20	0	30	0	0	0	0	0	0	0	100	-20	0	0	0	0	30	0	0
X_{13}	20	30	0	0	0	25	15	-15	0	20	0	-20	100	0	0	0	20	0	0	25
X_{14}	0	0	0	0	0	0	0	0	0	0	0	0	0	100	0	0	0	0	0	0
X_{15}	0	-20	0	0	0	0	0	0	0	0	0	0	0	0	100	0	0	0	0	0
X_{16}	0	0	0	0	0	0	0	0	0	0	0	0	0	0	0	100	0	0	0	0
X_{17}	20	0	20	0	0	0	0	-45	0	0	0	0	20	0	0	0	100	0	0	0
X_{18}	0	-15	0	45	20	0	0	-25	0	0	0	30	0	0	0	0	0	100	-40	0
X_{19}	-20	20	0	0	0	0	0	0	0	0	0	0	0	0	0	0	0	-40	100	0
X_{20}	0	45	0	0	0	45	25	0	0	30	0	0	25	0	0	0	0	0	0	100

Table S1. Correlation matrix multiplied by 100.
