# Supplementary material for: Evaluating variable selection methods for multivariable regression models: A simulation study protocol
Source: PLoS One. 2024 Aug 9;19(8):e0308543. doi: 10.1371/journal.pone.0308543 (PMC11315300; doi:10.1371/journal.pone.0308543)
Supplement: Attachment — Submitted filename: Response to reviewers.pdf [file pone.0308543.s006.pdf]

Evaluating variable selection methods for multivariable regression models: a simulation study protocol

Response to reviewers

We are grateful to the editor and the reviewer for their careful evaluation of our submission and their valuable comments. All comments are addressed below.

Comments of the editor

1. "As pointed out by the reviewer, the setting for the simulations is comparable simple compared to the real world cases, please address this issue seriously."
   The following corresponds to our answer to Comment 1 of the reviewer.
   We agree with the reviewer that variable selection in the (ultra)high-dimensional case is a highly relevant and interesting topic. Still, our focus in the proposed simulation study is somewhat different. Our setting with twenty variables reflects low-dimensional data. We have been involved in numerous collaborations with clinical researchers as can be seen from our departmental website. In our experience, low-dimensional data frequently appears in medicine and other application fields. While twenty variables may appear to be a relatively low number, researchers in medicine or other fields might still consider this number to be too high, and may wish to perform data-driven variable selection to reduce the number of variables in the final model. This could be for a number of reasons, for example:
   - the researchers suspect that not all twenty variables are relevant, but they do not have enough background knowledge to specify a priori which variables might be relevant;
   - a model with twenty coefficients is difficult to interpret, particularly when the goal of the modelling is *description* – i.e., finding a simple and parsimonious model (we mention this term in the Introduction, line 32);
   - it might be costly to collect all twenty variables (e.g., if collecting the variables requires cost-intensive examinations);
   - it is cumbersome and time-consuming to apply a model with twenty variables at the bedside in a clinical setting.

   Indeed, in many applied studies where variable selection was performed, the initial number of variables is comparable to our simulation setting. For example, consider regression models for COVID-19 prognosis. Wynants et al. 2020 performed a systematic review of such models. For the models where data-driven variable selection was applied, the median initial number of variables was 28 (details are given below in the paragraph in red colour).

To sum up, we think that our focus on the low-dimensional setting is practically relevant and reflects real-world data. To clarify that we do not consider the (ultra)high-dimensional case, we have added the following sentences in Section 1 (Introduction, lines 57-69):

Our focus is on low-dimensional data, which is reflected in our simulation setting with twenty independent variables. Data of this type frequently appears in medicine and other application fields, and researchers often apply variable selection in this context. For example, a systematic review of models for COVID-19 prognosis (Wynants et al. 2020) identified 236 newly developed regression models for prediction. Data-driven variable selection was applied (and reported) for 196 models. In 165 models both the number of candidate predictors (i.e., the predictors considered at the start of data-driven selection) and the number of predictors in the final model were reported; the median numbers were 28 (range 4 - 130), and 6 (range 1 - 38), respectively. This demonstrates that low- to medium-dimensional data played an important role in COVID-19 prediction research.

Of course, data-driven variable selection is also relevant for high-dimensional data. Comparing variable selection methods for high-dimensional data would require a different study design and is not the purpose of this planned simulation study.

Of course, evaluating variable selection methods in the (ultra)high-dimensional case would also be very relevant and could be an interesting topic for future research. We now mention this in Section 5 (Final Remarks, lines 529-530):

We focus on low-dimensional data in our study. Future studies could compare variable selection methods for high-dimensional data.

2. “Also, the authors mentioned when other relevant studies carried out a comparison between their proposed method and the existing methods, the number of the existing methods considered is very limited. However, the authors themselves have not carried out any comprehensive comparison with relevant methods. Please revise the corresponding paragraphs.”

   In the proposed simulation study, we do not to wish to introduce a new method and compare it to existing methods. Rather, as stated in Section 1 (Introduction, lines 13-31 and 70-87), this protocol outlines our plan to conduct a neutral comparison study of popular variable selection methods that were already introduced a while ago. We think that neutral comparison studies are vitally important because the performance evaluations in such studies are less biased than in studies which introduce new methods (this was pointed out, for example, in a PLOS ONE article by Boulesteix et al. 2013). The importance of neutral comparison studies has been increasingly acknowledged in recent years. For example, recently the *Biometrical Journal* released a special issue entirely dedicated to neutral comparison studies (see here). Because it is impossible to include all existing variable selection

methods in a single neutral comparison study, we selected the most popular of these methods. All in all, we plan to compare fourteen methods or method variants to each other, which is a sizable number:

1. forward selection with AIC
2. stepwise forward selection with AIC
3. backward elimination with $\alpha = 0.05$
4. backward elimination with $\alpha = 0.50$
5. backward elimination with AIC
6. backward elimination with BIC
7. augmented backward elimination (ABE) with AIC and $\tau = 0.05$
8. univariable selection with $\alpha = 0.05$
9. univariable selection with $\alpha = 0.20$
10. univariable selection with $\alpha = 0.20$ followed by background elimination with $\alpha = 0.05$
11. Lasso with λ tuned with 10-fold cross-validation
12. relaxed Lasso with λ tuned with 10-fold cross-validation
13. relaxed Lasso with λ tuned with BIC
14. adaptive Lasso with λ tuned with 10-fold cross-validation).

We have added one of these methods (method 10: univariable selection followed by backward elimination) after considering the reviewer's comments, see below. Of course, more methods could be considered in future studies (e.g., as briefly stated in Section 5, one might wish to compare Bayesian methods for variable selection in future research).

3. "Lastly, when I was a student and my professor told us that the coefficients before g(x) are still called linear coefficients, but the authors named them as nonlinear ones. Please double check it. If I am correct, please revise the corresponding equations and sentences."

   Thank you for pointing out this issue! We did not use the term „nonlinear coefficients", but indeed we denoted the coefficients for $g_j(X_j)$ as $\beta_j^{(NL)}$ (with "NL" for "nonlinear"), which might have been confusing. We use a new notation: $\beta_j^{(g)}$, where the letter g alludes to the nonlinear transformations g_j.

Comments of the reviewer

1. "Variable selection first come to notice in (ultra)high-dimensional (HD) cases. In the simulations, I do not see this is highlighted and thus the simulation cannot be a reflective of real-world problems and not practical. In the revision, the authors must exactly specify the relation between the sample size and dimension."

We agree with the reviewer that variable selection in the (ultra)high-dimensional case is a highly relevant and interesting topic. Still, our focus in the proposed simulation study is somewhat different. Our setting with twenty variables reflects low-dimensional data. We have been involved in numerous collaborations with clinical researchers as can be seen from our departmental website. In our experience, low-dimensional data frequently appears in medicine and other application fields. While twenty variables may appear to be a relatively low number, researchers in medicine or other fields might still consider this number to be too high, and may wish to perform data-driven variable selection to reduce the number of variables in the final model. This could be for a number of reasons, for example:

- the researchers suspect that not all twenty variables are relevant, but they do not have enough background knowledge to specify a priori which variables might be relevant;
- a model with twenty coefficients is difficult to interpret, particularly when the goal of the modelling is *description* – i.e., finding a simple and parsimonious model (we mention this term in the Introduction, line 32);
- it might be costly to collect all twenty variables (e.g., if collecting the variables requires cost-intensive examinations);
- it is cumbersome and time-consuming to apply a model with twenty variables at the bedside in a clinical setting.

Indeed, in many applied studies where variable selection was performed, the initial number of variables is comparable to our simulation setting. For example, consider regression models for COVID-19 prognosis. Wynants et al. 2020 performed a systematic review of such models. For the models where data-driven variable selection was applied, the median initial number of variables was 28 (details are given below in the paragraph in red colour).

To sum up, we think that our focus on the low-dimensional setting is practically relevant and reflects real-world data. To clarify that we do not consider the (ultra)high-dimensional case, we have added the following sentences in Section 1 (Introduction, lines 57-69):

Our focus is on low-dimensional data, which is reflected in our simulation setting with twenty independent variables. Data of this type frequently appears in medicine and other application fields, and researchers often apply variable selection in this context. For example, a systematic review of models for COVID-19 prognosis (Wynants et al. 2020) identified 236 newly developed regression models for prediction. Data-driven variable selection was applied (and reported) for 196 models. In 165 models both the number of candidate predictors (i.e., the predictors considered at the start of data-driven selection) and the number of predictors in the final model were reported; the median numbers were 28 (range

4 - 130), and 6 (range 1 - 38), respectively. This demonstrates that low- to medium-dimensional data played an important role in COVID-19 prediction research.

Of course, data-driven variable selection is also relevant for high-dimensional data. Comparing variable selection methods for high-dimensional data would require a different study design and is not the purpose of this planned simulation study.

Of course, evaluating variable selection methods in the (ultra)high-dimensional case would also be very relevant and could be an interesting topic for future research. We now mention this in Section 5 (Final Remarks, lines 529-530):

We focus on low-dimensional data in our study. Future studies could compare variable selection methods for high-dimensional data.

2. “In this paper, I did not see any trace of screening. Why did not authors consider selecting important variables based on the marginal correlation of conduct sure screening?”

   In our list of methods that we plan to compare, univariable screening is indeed included (with two different thresholds, namely $\alpha = 0.05$ and $\alpha = 0.20$). We call this “univariable selection” instead of “univariable screening”, but we do mean the same method (i.e., evaluating the association of each variable with the outcome by fitting univariable regression models; for linear regression, this is equivalent to calculating the marginal correlations between the independent variables and the outcome).

   Other screening methods could also be considered, but these might be more relevant for the (ultra)high-dimensional case, which we do not focus on (see the answer to point 1).

   Still, the reviewer’s comment inspired us to include another method: since univariable screening followed by backward elimination is frequently applied in practice, we have decided to add this method to our comparison study (more precisely, univariable selection with $\alpha = 0.20$ followed by background elimination with $\alpha = 0.05$).

3. “The problem of multicollinearity is not considered in variable selection which is absolutely important.”

   We agree that multicollinearity is a very important topic in the context of variable selection. Of course, data-driven variable selection methods tend to perform worse if there is a high degree of correlation between the predictors, and their performance will improve the less the predictors are correlated with each other. Before regression analysis is performed in an applied study, the correlations between the independent variables should be checked during initial data analysis (Heinze et al. 2023, preprint). The consequence of multicollinearity also depends on the purpose of analysis (Gregorich et al 2021, link to paper); we do not advocate to just let a data-driven variable selection method decide how to handle multicollinearity. For our simulation study, we have carefully chosen true correlations between the independent variables based on a real correlation matrix from NHANES data. The correlations of the independent variables with each other are demonstrated in the supplemental

figure S2, which shows the coefficients of determination R_j^2 for the regression of each variable X_j on all other respective variables $X_l, l = 1, \dots, 20, l \neq j$. The R_j^2 values range from 0 to 0.56, demonstrating differing degrees of dependence between the predictors. The maximum R_j^2 value of 0.56 corresponds to a multiple correlation coefficient of 0.75, which is already quite high. These values will be considered when interpreting the simulation results. In the supplemental figure S1, we show the network of the *pairwise* correlations of the variables, and in the supplemental table S1, the correlation matrix. It can be seen that the pairwise correlations between the predictors are at most 0.45. In our experience, higher pairwise correlations appear when there is a systematic relation between predictors (e.g., consider the two variables "previous diagnosis of hypertension - yes/no" and "patient takes medication against hypertension - yes/no"); in that case, it might be questionable if these related variables should be selected independently using a data-driven variable selection algorithm. This is why we do not consider pairwise correlations higher than 0.45.

To emphasize the importance of multicollinearity in the context of variable selection, we have added the following sentences in Section 5 (Final remarks, lines 503-514):

Multicollinearity is an important topic in the context of variable selection. Data-driven variable selection methods tend to perform worse if there is a high degree of correlation between the predictors, and their performance will improve the less the predictors are correlated with each other. Before regression analysis is performed in an applied study, the correlations between the independent variables should be checked during initial data analysis (Heinze et al. 2023). For our simulation study, we have carefully chosen true correlations between the independent variables based on a real correlation matrix from NHANES data. As mentioned in Section 3.2.1, S2 Fig shows the coefficients of determination R_j^2 for the regression of each variable X_j on all other respective variables $X_l, l = 1, \dots, 20, l \neq j$. The R_j^2 values range from 0 to 0.56, demonstrating differing degrees of dependence between the predictors. These values will be considered when interpreting the simulation results.

4. "The measure R-squared is not a good measure of model fit in HD problems."

   We agree that R^2 might not be a good choice if it is used as a performance measure to evaluate a fitted model; alternatives such as the adjusted R^2 might be preferable. However, in our study, we do not use R^2 as a performance measure to evaluate the fitted models obtained with variable selection. That is, R^2 is not in the list of performance measures as given in Section 3.5 and S1 Appendix. Instead, we use R^2 to describe our data-generating mechanisms. The R^2 values that are given in the protocol (e.g., in Table 1) refer to the R^2 values of the "true" models (i.e., the population models). We think that R^2 is a suitable

measure for describing the association of the predictors with the outcome in the simulated population.

5. “Form of explanatory variables does not play substantial role; however, the dependent variable plays. I do not see variant responses. Furthermore, in such studies, it is of essential need to consider additive structures in the predictive component. Further investigations for multivariate additive models are needed.”

   In application fields such as medicine, the distributions of the explanatory variables are often relevant. Variables can have different types and distributional forms. Since we want to make the simulated data as realistic as possible, this is reflected in our simulation design: we have a mixture of binary variables and continuous variables; for the continuous variables, we consider both symmetric and skewed distributions, as is often seen in practice.

   As for the dependent variable, we consider two different types: continuous (for linear regression) and binary (for logistic regression). These response types are very relevant in applications. Considering linear and logistic regression means that we already consider additive models. As mentioned in Section 5 (Final Remarks, lines 518-519), we plan to consider other response types (e.g., survival times) in future research.

   In the present protocol, we have also included settings with mildly misspecified functional forms (nonlinear effects). That is, we consider the consequences of fitting models that assume linear functional forms of continuous predictors, even though the true functional forms are nonlinear. As noted in Section 5 (Final remarks, lines 527-528), future simulation studies should include models that take nonlinear effects into account (generalized additive models), but this goes beyond the scope of our current study.
